# Unexpected Dramatic Evolution of Placenta Increta: Case Report and Literature Review

**DOI:** 10.3390/jpm13111563

**Published:** 2023-10-31

**Authors:** Mihaela Camelia Tîrnovanu, Vlad Gabriel Tîrnovanu, Bogdan Toma, Loredana Toma, Elena Țarcă, Laura Stătescu, Ștefan Dragoș Tîrnovanu, Carmen Ungureanu, Mioara Florentina Trandafirescu, Jana Bernic, Elena Cojocaru

**Affiliations:** 1 Department of Mother and Child Medicine, “Grigore. T. Popa” University of Medicine and Pharmacy, 700115 Iasi, Romania; mihaela.tirnovanu@umfiasi.ro (M.C.T.); vlad-gabriel.cl.tirnovanu@students.umfiasi.ro (V.G.T.); loredana-toma@umfiasi.ro (L.T.); 2 “Cuza Voda” Obstetrics-Gynecology Clinic Hospital, 700038 Iasi, Romania; bogdan.f.toma@umfiasi.ro; 3 Department of Morphofunctional Sciences I, “Grigore T. Popa” University of Medicine and Pharmacy, 700115 Iasi, Romania; carmen.ungureanu@umfiasi.ro (C.U.); mioaratrandafirescu@yahoo.co.uk (M.F.T.); elena2.cojocaru@umfiasi.ro (E.C.); 4 Department of Surgery II, “Grigore T. Popa” University of Medicine and Pharmacy, 700115 Iasi, Romania; stefan-dragos.tirnovanu@umfiasi.ro; 5 Department of Dermatology, “Grigore T. Popa” University of Medicine and Pharmacy, 700115 Iasi, Romania; 6 Discipline of Pediatric Surgery, “Nicolae Testemițanu” State University of Medicine and Pharmacy, 2025 Chisinau, Moldova; jana.bernic@usmf.md

**Keywords:** postpartum hemorrhage, placenta increta, cesarean section

## Abstract

Placental morbid adherence is a known risk factor for postpartum hemorrhage. The incidence of abnormal placental attachment has been increasing over the past few decades, mainly due to rising rates of cesarean deliveries, advanced maternal age, and the use of assisted reproductive technologies. Cesarean section is a significant risk factor for placenta increta, as it disrupts the normal architecture of the uterine wall, making it more difficult for the placenta to detach after delivery. We present the case of a woman who underwent a cesarean section at 28 weeks due to anterior placenta previa, accompanied by hemorrhage and rupture of membranes. Following the delivery, she experienced normal postoperative bleeding and was discharged home after five days. However, six weeks later, she presented with heavy bleeding, leading to the decision to perform a total hysterectomy. The levels of HCG were found to be low. The pathological examination of the specimens confirmed a diagnosis of placenta increta, as it revealed notable placental proliferation, necrotic villi, and placental invasion near the uterine serosa. Notably, we did not find any similar cases documented in the literature. Patients experiencing prolonged vaginal bleeding after childbirth and diagnosed with placenta accreta should be closely monitored through ultrasound examinations; abnormal proliferation of the placenta can occur, and prompt detection is crucial for appropriate management.

## 1. Introduction

Placental morbid adherence, a form of abnormal placentation, is a serious and potentially life-threatening condition that occurs when the placenta attaches too deeply and firmly to the uterine wall. This abnormal attachment can result in significant complications during pregnancy, delivery, and the postpartum period. Over the years, our knowledge of morbid adherence of the placenta has greatly expanded, leading to improved diagnosis, management, and outcomes for affected patients [1].

Research efforts have focused on improving our understanding of the pathophysiology, risk factors, and prevention strategies for morbid adherence and decreased fertility. Risk factors such as previous cesarean sections, placenta previa, advanced maternal age, prior uterine surgeries, chemicals, and socio-epidemiological factors have been established, aiding in the identification of high-risk patients who may benefit from targeted interventions or specialized care [2,3].

Morbid adherence of the placenta represents a significant risk factor for postpartum hemorrhage (PPH). PPH is defined as excessive bleeding following childbirth, typically within 24 h after delivery. When the placenta is abnormally attached to the uterine wall and invades the myometrium, it becomes difficult to detach naturally during the third stage of labor. This can result in retained placental tissue and impaired contraction of the uterus, leading to postpartum bleeding [1].

When bleeding begins more than 24 h after birth, it is known as delayed postpartum hemorrhage and is generally acknowledged that retained placenta is one of the main causes [4]. Retained placenta is more prevalent in developed nations, probably because of more frequent treatments such as therapeutic abortion (for miscarriage or chromosomal abnormalities) or labor induction, yet mortality is quite low [5]. If the retained placental fragments are small, they may spontaneously resorb, in which case conservative or hormonal therapy may be successful [6].

Recent guidelines suggest using the term “placenta accreta spectrum” (PAS) to encompass these conditions [7]. Although a placenta previa can result in bleeding following a cesarean operation, the examination of the exposed uterus allows for a thorough assessment and the manual removal of the adherent placenta fragment. Severe vaginal bleeding, cesarean hysterectomy, and blood transfusion during pregnancy may be caused by abnormal placenta attachment to the myometrium [8].

The prevalence of cesarean deliveries has been rising in recent years, which has led to an increase in the incidence of placenta accreta, a rare and life-threatening complication of pregnancy, as well as other complications [2]. The most accepted hypothesis explains that PAS occurs in a scarred uterus. Following uterine scarring, there is a failure in the normal decidualization of the area, causing a secondary defect in the endometrial–myometrial junction. Placenta accreta develops because of decidua basalis’s total or partial absence, resulting in placental anchoring villi coming in direct apposition with the myometrium due to a deficient Nitabuch’s layer [9].

A recent study has shown that following a cesarean section, uterine vascular resistance is increased, which in turn decreases the blood flow globally in comparison to that following a normal vaginal delivery [10]. The inadequate vascularization of the scar tissue endangers re-epithelialization and leads to the emergence of permanent focal myometrial degeneration. At the level of poorly epithelialized scar tissue, trophoblasts invade through the decidua and myometrium, reaching the underlying blood vessels and surrounding structures. Trophoblast cellular changes, probably secondary to the rare myometrial condition leading to excessive invasion into the myometrium, were detected in PAS. [11].

In a perfect world, obstetrical ultrasound results would trigger the initial suspicion of placenta accreta while the patient remains asymptomatic [12]. Usually, patients present with immediate postpartum hemorrhage, requiring a hysterectomy. For the placenta’s anterior location, the degree of invasion can be established using magnetic resonance imaging (MRI) or ultrasound [13,14].

Only a few publications with cases of late vaginal bleeding from the placental site caused by necrotic villi are found in the literature and usually, they present with only a zone of damaged myometrium caused by placental invasion [15] after abortion or vaginal birth and no proliferation of the placental tissue. The management of these cases is difficult. Two cases of placenta increta that resulted in prolonged vaginal bleeding and uterine mass have been documented, however, they were both following first-trimester abortions [9].

## 2. Case Report

We present the case of delayed postpartum bleeding in a 36-year-old patient with regular prenatal follow-up from the first trimester. The diagnosis of placenta previa was established early in the pregnancy as a marginal one, but the pregnancy was not followed up in our hospital. She did not receive an MRI during pregnancy for this diagnosis. At 28 weeks of gestation, she presented in the emergency department with significant vaginal bleeding, premature rupture of membranes, and chorioamnionitis. The diagnosis of chorioamnionitis was established before the caesarean section based on a laboratory analysis showing a leukocyte count of 19,980 and C-reactive protein in the amount of 50.2 mg/L (laboratory limit-5 mg/L). It is noteworthy that she had a previous cesarean section a decade ago. Through ultrasonography, we identified that she had anterior lateral placenta previa, located 2 cm from the internal os of the cervix. Consequently, a lower transverse cesarean section was performed (in the same manner as the previous one), and a boy in a cephalic presentation, weighing 1500 g, was delivered via transplacental extraction. Following the extraction of the fetal placenta and membranes, we discovered a small area of placental attachment on the posterior part of the uterine segment near the incision, with increased adhesion, and an otherwise clean uterine cavity. These small placental fragments were successfully removed, ensuring effective hemostasis. Subsequent histologic examination of the placenta revealed characteristics which can indicate placenta accreta (chorionic villi implanted directly on the surface of the myometrium without intervening decidua; fibrin and extravillous trophoblasts present between the villi and myometrial fibers) and chorioamnionitis.

The patient had a favorable postoperative recovery with normal vaginal bleeding, leading to her discharge after five days. No immediate postpartum ultrasound was performed because the patient had a normal evolution in the first postoperative days, without quantitatively abnormal vaginal bleeding and with normal uterine involution. From hospital discharge, she visited the hospital daily to breastfeed her baby until the infant reached a weight of 2500 g, at which point both mother and baby were able to go home. However, later on, she experienced a significant episode of delayed postpartum hemorrhage, occurring six weeks after giving birth. The patient reported an intermittent small amount of vaginal bleeding during this period. During the clinical bimanual examination, an enlarged uterus of approximately 12–13 cm with a soft consistency was detected.

**Figure 1:**
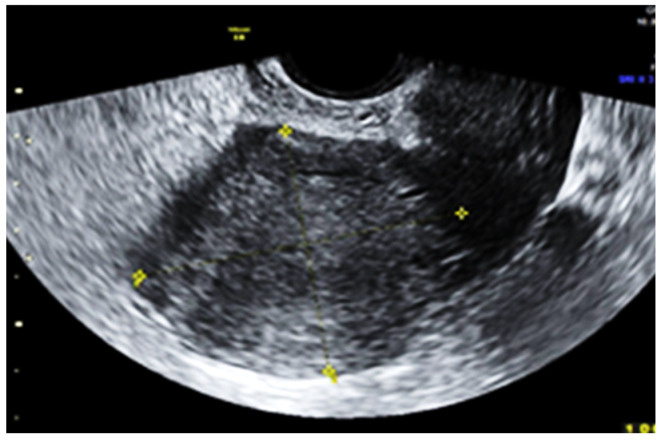
The normal aspect of the uterine body.

**Figure 2:**
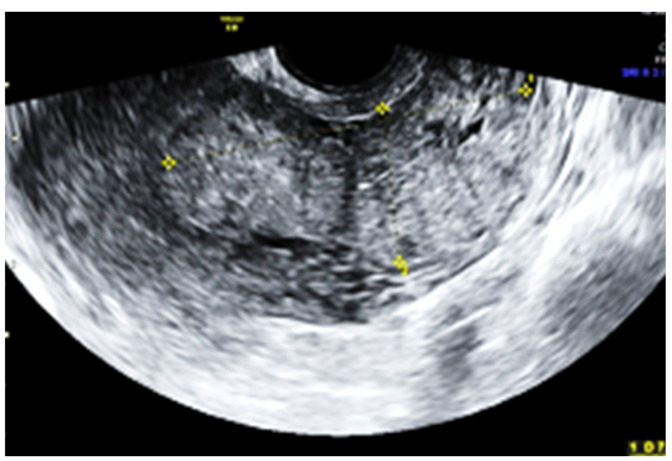
The mass located in the inferior part of the uterus and the upper part of the cervical channel.

**Figure 3:**
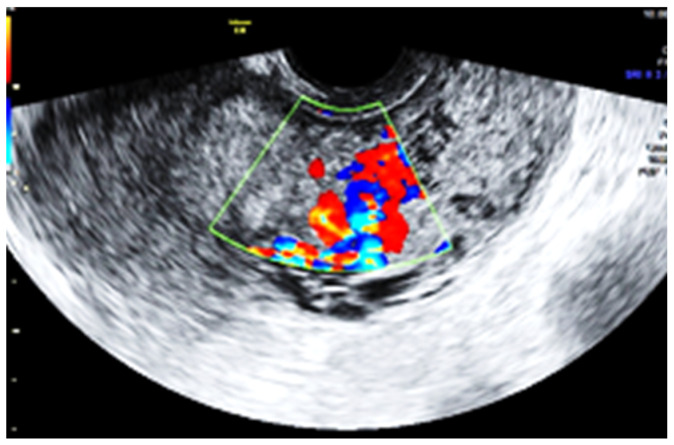
The mass with increased vascularity.

**Figure 4:**
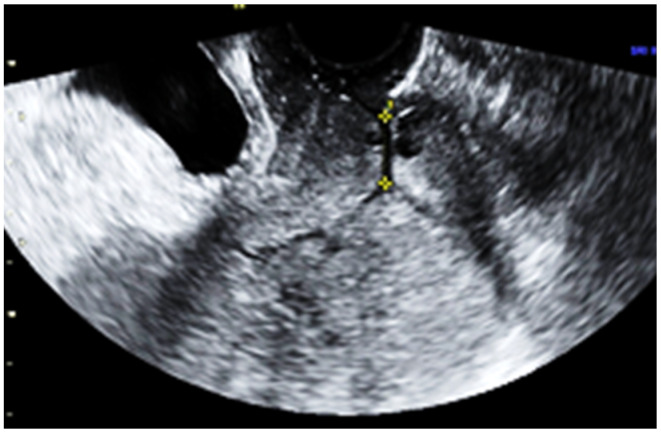
The lower part of the uterine cervix with normal characteristics.

Subsequent ultrasound examination revealed a uterus with a homogeneous structure measuring 67/52/61 mm (Figure 1). However, in the lower portion of the uterine cavity and the upper part of the cervical canal, there was a non-homogeneous hyperechogenic mass measuring 72/30/75 mm (Figure 2). The mass exhibited numerous vessels (Figure 3), while the lower portion of the cervix appeared normal with a length of approximately 13 mm (Figure 4).

Based on the ultrasound findings, we suspected a diagnosis of choriocarcinoma originating from the placental site. The patient had a low beta-HCG level (15.5 IU), and an MRI confirmed that the uterine mass consisted solely of placental tissue, without the involvement of the uterine walls or adjacent organs.

**Figure 5:**
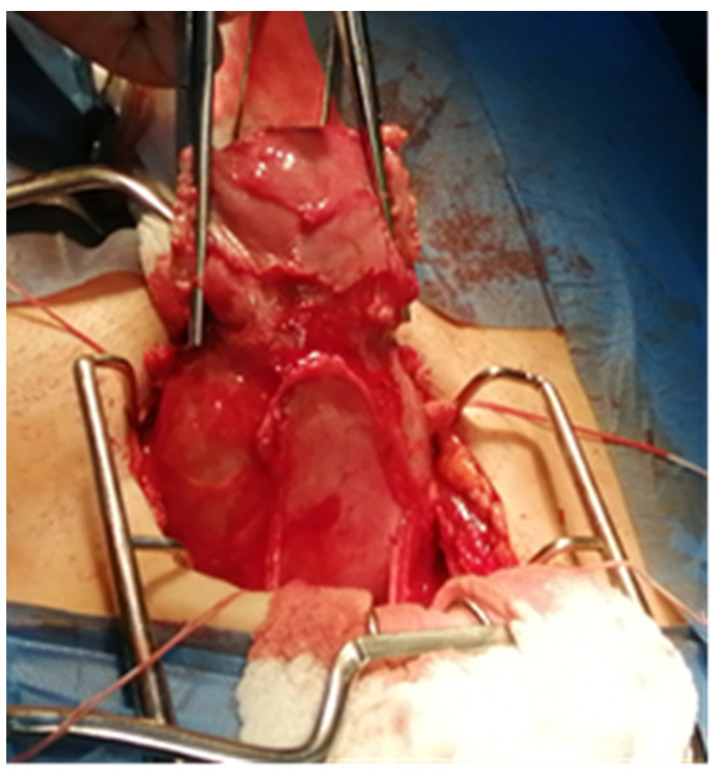
The mass is located in the inferior part of the uterus.

**Figure 6:**
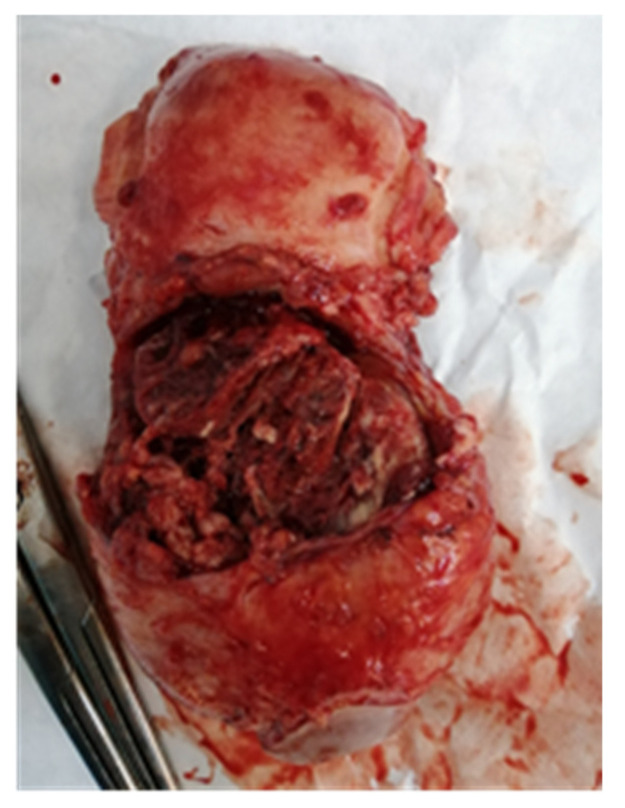
Posterior rupture of the uterus with visualization of the mass.

Subsequently, the patient was readmitted to the hospital. Responding to her request, we attempted a conservative treatment approach by performing curettage under ultrasound guidance. Frozen sections taken during the procedure did not indicate any evidence of trophoblastic disease—it was normal placental tissue. However, during the maneuver, we observed the increased adhesion of the proliferated placental tissue to the posterior uterine wall. Unfortunately, due to the occurrence of massive bleeding during the curettage, a total hysterectomy was necessary. Additionally, to provide adequate compression, three vaginal meshes were introduced until the uterus was removed. During the surgery, the uterus was found to be extremely fragile (Figure 5), with a posterior rupture at the isthmus level (Figure 6), where the placental invasion extended up to the uterine serosa.

The PAS grading is a pathologic grading system based on the gross and microscopic evaluation of the hysterectomy specimen. Grade 3A (according to the new grading system) was suspected; the mass was developed posteriorly and not anteriorly towards the urinary bladder. The mass, consisting of retained placental tissue with degenerated villi, fibrin, and inflammatory exudate, extended in the uterine cavity and the cervical canal. The uterine rupture occurred through two intricate mechanisms: the placenta invaded and weakened the posterior wall of the uterus; and, at the time of the posterior dissection of the uterus and due to traction, the uterine wall ruptured.

**Figure 7:**
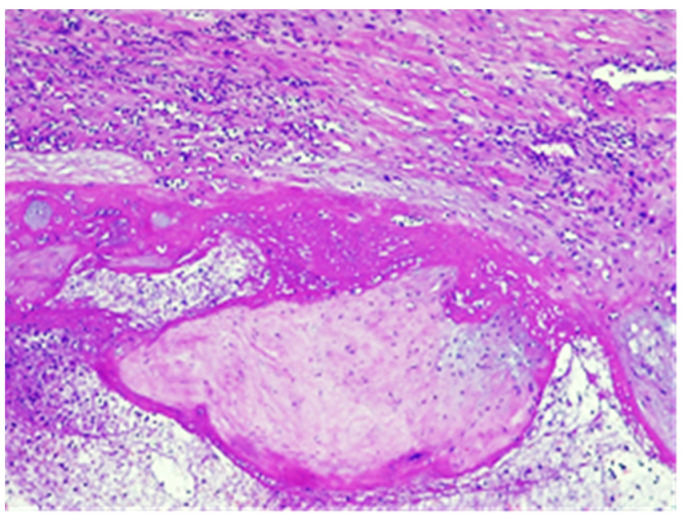
Chorionic villus without decidua in the thickness of the myometrium, hematoxylin and eosin stain, ×10.

**Figure 8:**
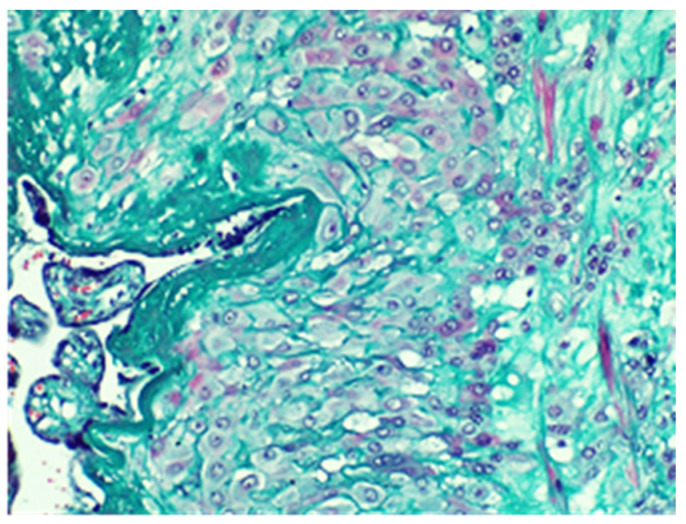
Placenta with aspects of accretion-muscle fibers colored in red on the right of the image, Van Gieson stain, × 20.

**Figure 9:**
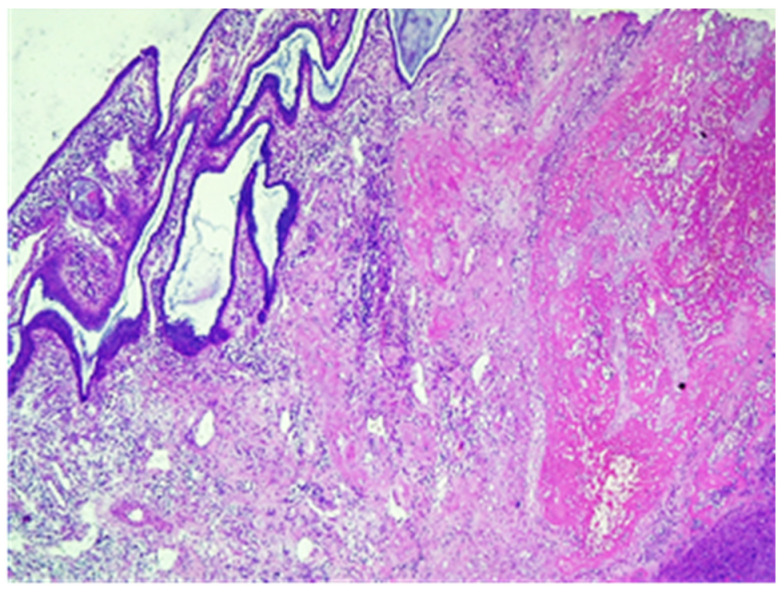
Chorionic villi in the endocervical region, in the left endocervical mucosa, hematoxylin and eosin stain, × 4.

**Figure 10:**
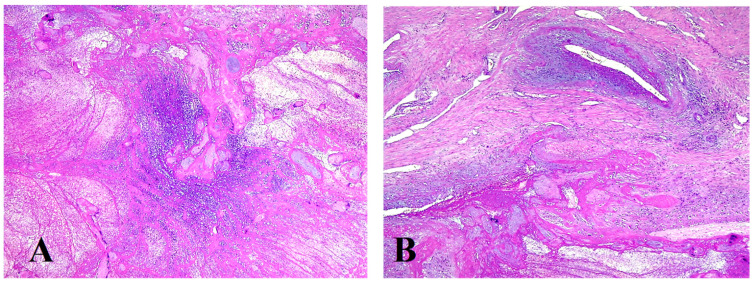
Necrotic chorial villi (**A**) included in fibrino-leucocytic exudate (**B**) in the vicinity of the large vessels in the external half of the wall, hematoxylin and eosin stain, × 4.

The conclusive histological examination demonstrated placenta increta (Figure 7 and Figure 8) within the uterine cavity and cervical canal (Figure 9). Degenerative necrotic changes (Figure 10A,B) and acute inflammation were also observed.

The patient experienced a positive postoperative recovery and was discharged after five days. We monitored her HCG levels at 1, 3, 6, 9, and 12 months, and they remained consistently low, measuring less than 2 IU.

## 3. Discussion

The severity of postpartum hemorrhage in cases of morbid placental adherence can be quite significant. The risk of PPH is higher in cases of placenta increta compared to other forms of abnormal placental attachment, such as placenta accreta or placenta previa. The risk is further increased in cases of placenta percreta, where the placenta penetrates through the full thickness of the uterine wall and can even invade nearby organs [16].

Placenta accreta is a pregnancy complication that poses a significant risk to the life of the mother. It occurs when there is a complete or partial absence of the decidua basalis and imperfect development of the fibrinoid layer (Nitabuch layer) [17,18]. 

The interactions between the maternal–fetal interfaces are believed to play a role in its development. Several risk factors have been associated with abnormal placental attachment, including placenta previa, previous cesarean section, previous uterine curettage, multiparity (≥6), advanced maternal age, and surgeries such as adenomyomectomy [19] or myomectomy for submucosal myoma. The patient in our current report presented only one risk factor, previous cesarean sections, respectively (and dilation and curettage procedures).

Depending on the depth of placental penetration into the uterine wall, this condition can be further classified into placenta accreta (where chorionic villi directly implant onto the myometrium), placenta increta (where chorionic villi invade into the myometrium), and placenta percreta (where chorionic villi invade through the myometrium and may involve surrounding structures). Recent guidelines suggest using the term “placenta accreta spectrum” (PAS) to encompass these conditions [7].

The grades of PAS invasion and local tissue destruction are designated as follows: PAS Grade 1—noninvasive: adherent placenta—there is a smooth placental–myometrial interface and uniform myometrial thickness, PAS Grade 2—superficial invasion: irregular placental–myometrial interface, with preservation of 25% of the wall thickness relative to the uninvolved myometrium, PAS Grade 3A—deep invasion: cross sections show involvement of the outer myometrium, but with intact serosa, PAS Grade 3D—deep invasion with disruption of the serosa, and PAS Grade 3E, extra uterine invasion (3D plus adherent extra uterine structures like the bladder or extra uterine fibroadipose tissue) [20].

Placenta increta is typically characterized by difficult placental removal during childbirth and severe vaginal bleeding in the third trimester. However, it can also present with post-curettage hemorrhage in the first and second trimesters. In many cases, patients may not experience preceding symptoms, and early diagnosis relies on a high level of suspicion and recognition of the associated risk factors [21].

Placenta increta has been reported to present with various clinical manifestations in the first trimester of pregnancy. Previous reports have described cases of uterine rupture [14], immediate and massive acute bleeding following dilation and curettage [22], persistent vaginal bleeding after curettage [23], intraperitoneal bleeding after uncomplicated first-trimester dilation and curettage, and the presence of a uterine mass [24]. Ju et al. reported the first case of placenta increta presenting as a uterine mass two weeks after a first-trimester abortion, with magnetic resonance imaging revealing a mass within the myometrium [25]. Our patient had light intermittent vaginal bleeding after discharge from the hospital until the heavy bleeding six weeks after the caesarean section, which is why she came for consultation. With the uterus open during the cesarean section (lower segment transverse incision), the surface of the uterine cavity was completely clean and was not bleeding profusely from anywhere. The incision was low enough to inspect the lower part of the uterus and the cervical canal. Such a large placental growth with no visible post-cesarean residual placenta was surprising. The curettage was performed at the patient’s insistence, although it was explained to her that there would be heavy bleeding, and ultimately, she would need to undergo a hysterectomy. When opening the abdomen, the significant mass in the lower part of the uterus that infiltrated the myometrium up to the serosa was visible. Opening the uterine cavity was not warranted as it was not possible to remove just the placental tissue. Lim et al. reported a similar case where a uterine mass developed three years after dilation and curettage for a first-trimester abortion. Severe vaginal bleeding during curettage led to an emergency hysterectomy, and pathology findings confirmed placenta accreta [26].

Several comparisons can be drawn between the microenvironment of the placenta accreta spectrum and tumor behavior. Both conditions require the ability of cells to evade the local immune system, induce angiogenesis, and promote invasion [27]. This may explain the proliferation of placental tissue even though the uterine cavity appeared normal after placental removal during the cesarean section. Extensive neovascularization is a prominent feature in most PAS cases, as observed in the increased vascularity of the intrauterine mass detected during our patient’s ultrasound examination. Comparative analysis of syncytiotrophoblastic cells from PAS cases and normal placenta specimens has suggested a proangiogenic phenotype in PAS [28].

The clinical history, radiologic findings, and elevated HCG levels are crucial for obstetricians to diagnose abnormalities related to retained placenta [29,30]. In our case, however, the patient presented with a low HCG value of only 15.5 U. Our patient underwent MRI to rule out choriocarcinoma (although beta HCG was low), and also to evaluate the local extrauterine extension of the mass. Postpartum choriocarcinoma is a very rare complication of pregnancy [31]. The correct diagnosis is only made with pathological examination and the prognosis for women with choriocarcinoma after non-molar pregnancies is worse, due to delay in diagnosis or advanced disease [31,32]. The differential diagnosis of placenta increta must also consider placental site trophoblastic tumor, epithelioid trophoblastic tumor, exaggerated placental site, and atypical placental site nodule [31,32,33,34,35]. Placental site trophoblastic tumor is a rare form of gestational trophoblastic disease and represents a malignant transformation of intermediate trophoblastic cells. It occurs several months, or even years after an abortion, term delivery of a normal pregnancy, or after ectopic or molar pregnancy [33]. Placental site trophoblastic tumors and epithelioid trophoblastic tumors develop in the area where the placenta joins the lining of the uterus. Pathological examination is obligatory for confirmation of the exact disease.

Managing postpartum hemorrhage in cases of morbid placental adherence requires a coordinated approach involving obstetricians, anesthesiologists, and urologists if there exists an invasion of the urinary bladder. When considering options for preserving the uterus through medical or conservative surgical therapy, such as uterine artery embolization, it is essential to take this differential diagnosis into account before proceeding with a second surgery [36,37]. In our case, although uterine artery embolization could have been a potential option to control vaginal bleeding, the size of the mass led us to conclude that surgical removal was necessary.

Early detection and appropriate management are crucial in preventing complications associated with postpartum hemorrhage. Advancements in diagnostic techniques have played an important role in identifying cases of morbid adherence. Prenatal ultrasound examinations, particularly specialized imaging techniques like magnetic resonance imaging (MRI) have become valuable tools in visualizing the placental location, depth of invasion, and involvement of adjacent structures. As such, they can help plan for the safest delivery method and potential interventions [14,38]. In fact, ultrasound is the preferred method for diagnosing abnormally invasive placenta during pregnancy, and its diagnostic accuracy has been demonstrated to be reliable in both retrospective and prospective studies [39].

The management of morbid adherence has also evolved over time. Previously, the primary approach involved a hysterectomy, which resulted in the loss of fertility for the patient. However, more conservative and minimally invasive approaches have been developed in recent decades to preserve the uterus, the ovaries, and fertility whenever possible [40]. Techniques such as uterine artery embolization, which involves blocking the blood supply to the placenta, have been utilized to control bleeding and facilitate safer removal of the placenta [41]. Additionally, interventions such as methotrexate administration and curettage under ultrasound guidance have been employed in selected cases [42].

Our patient requested conservative treatment and was offered the option of using methotrexate, but she refused. The duration during which methotrexate causes placental resorption and decreased vascularity is individual (differs from one patient to another) as in the case of cervical ectopic pregnancy or implanted on the post-cesarean scar. The number of doses of MTX administered depends on the evolution of the case, evaluated by ultrasound examination, the size of the mass of tissue, and the intensity of its vascularization. There are cases reported in the literature indicating conservative treatment with methotrexate for placenta accreta not extracted from the uterus during cesarean delivery; these cases involved an incision at the bottom of the uterus to extract the fetus, or total retention of the placenta after natural birth. The mechanism of action of methotrexate is to hasten placental resolution. The dose of methotrexate is 1 mg per meter square weekly, intramuscularly [43,44,45,46].

The rate of placental cell division in the third trimester is low compared to the first trimester, and this raises the question of whether methotrexate should be used for placental resorption. On the other hand, methotrexate can cause serious harm such as neutropenia and medullary aplasia, even with a single dose in a young patient; that is why the benefits of methotrexate should be weighed against the possible drug toxicity [47]. These adverse effects are particularly morbid if an infection appears, a common complication of conservative management [48]. In Table 1 we summarized some publications reporting similar cases, and the applied treatment.

Unfortunately, our attempt to remove the placenta using curettage was unsuccessful in this particular case, and we had to resort to performing a hysterectomy due to severe vaginal bleeding. The option of administering methotrexate was not feasible as the patient expressed a desire to continue breastfeeding.

When an abnormally invasive placenta is detected during the antenatal period, the risk of maternal mortality caused by placenta accreta spectrum is minimized as it enables early intervention and effective management. Placenta previa patients who develop PAS have shown a correlation with increased placental thickness, as indicated by various retrospective studies. The exact cause of this association remains uncertain, but it may be attributed to the placenta’s restricted migration resulting from its attachment to the cesarean scar defect, leading to a mushroom-like bulging of the placenta beyond the defect [42].

## 4. Conclusions

Patients experiencing prolonged vaginal bleeding after childbirth and diagnosed with placenta accreta should be closely monitored through ultrasound examinations. This is because an abnormal development of the placental tissue can occur, and prompt detection is crucial for appropriate management.

## Figures and Tables

**Table 1:** The main publications reporting similar cases of PAS and treatment methods.

Author	Number of Cases	Placental Pathology	Clinical Manifestation	Treatment
Cramer et al., 2019 [14]	3	placenta increta	retained placenta; delayed postpartum hemorrhage	hysterectomy for menorrhagia
Rouholamin et al., 2014 [15]	2	placenta increta	prolonged vaginal bleeding	curettage followed by hysterectomy; total abdominal hysterectomy
Lim et al., 2013 [25]	1	placenta accreta	vaginal bleeding three years after a first trimester abortion	total abdominal hysterectomy
Liu et al., 2003 [40]	4	placenta increta	severe hemorrhage in induced abortion in the first trimester	uterine artery embolization
Gregoir et al., 2022 [43]	5	placenta accreta spectrum	prolonged vaginal bleeding	methotrexate administration with or without embolization

## Data Availability

Not Applicable.
